# Staff’s normative attitudes towards coercion: the role of moral doubt and professional context—a cross-sectional survey study

**DOI:** 10.1186/s12910-017-0190-0

**Published:** 2017-05-25

**Authors:** Bert Molewijk, Almar Kok, Tonje Husum, Reidar Pedersen, Olaf Aasland

**Affiliations:** 1 Centre for Medical Ethics, Institute of Health and Society, University of Oslo, Oslo, Norway; 2 Department Medical Humanities, EMGO+, VU University medical centre (VUmc), Amsterdam, The Netherlands; 3 Department Epidemiology & Biostatistics, EMGO+, VU University medical centre (VUmc), Amsterdam, The Netherlands; 4 Institute for Studies of the Medical Profession, Oslo, Norway

**Keywords:** Coercion, Critical reflection, Epistemic uncertainty, Ethics, Ethics support, Mental health care, Moral case deliberation, Moral doubt, Normative attitude

## Abstract

**Background:**

The use of coercion is morally problematic and requires an ongoing critical reflection. We wondered if not knowing or being uncertain whether coercion is morally right or justified (i.e. experiencing moral doubt) is related to professionals’ normative attitudes regarding the use of coercion.

**Methods:**

This paper describes an explorative statistical analysis based on a cross-sectional survey across seven wards in three Norwegian mental health care institutions.

**Results:**

Descriptive analyses showed that in general the 379 respondents a) were not so sure whether coercion should be seen as offending, b) agreed with the viewpoint that coercion is needed for care and security, and c) slightly disagreed that coercion could be seen as treatment. Staff did not report high rates of moral doubt related to the use of coercion, although most of them agreed there will never be a single answer to the question ‘What is the right thing to do?’.

Bivariate analyses showed that the more they experienced general moral doubt and relative doubt, the more one thought that coercion is offending. Especially psychologists were critical towards coercion. We found significant differences among ward types. Respondents with decisional responsibility for coercion and leadership responsibility saw coercion less as treatment. Frequent experience with coercion was related to seeing coercion more as care and security.

**Conclusions:**

This study showed that experiencing moral doubt is related to some one’s normative attitude towards coercion. Future research could investigate whether moral case deliberation increases professionals’ experience of moral doubt and whether this will evoke more critical thinking and increase staff’s curiosity for alternatives to coercion.

*‘I know that I know nothing’*

Socrates

## Background

It is generally understood that the use of coercion is morally problematic and that health care professionals needs to critically reflect upon and morally justify the use of coercion. Internationally, many health care institutions are trying to reduce the use of coercion, find better alternatives for coercion and improve the way coercion is being executed. Being uncertain whether the use of coercion is morally justified (i.e. moral doubt) is often a starting point for critical reflection and changing cultures and attitudes towards coercion. Within this study we wanted to find out whether experiencing moral doubt is related to the normative attitudes of health care professionals towards coercion. A better understanding of the possible role of moral doubt in stimulating critical reflection and changing cultures might inform us whether stimulating moral doubt, for example by means of moral case deliberation, can play a role in reducing and improving the use of coercion. More in general, we think the role of moral doubt and critical reflection in improving practices needs further attention in both clinical practice and research.

### Research questions

1. What are mental health care professionals’ normative attitudes towards coercion and how often do they experience moral doubt?
2. How is health care professionals’ experienced moral doubt related to their normative attitude towards coercion?
3. How are professional and contextual characteristics related to the staff’s normative attitude towards coercion and to experiencing moral doubt?

### Coercion as morally problematic

The use of coercive measures in mental health care remains morally controversial and is often hotly debated. Even though there is a general understanding that coercive measures are inherently morally problematic and should be used as little as possible [1–3], it remains difficult to determine whether a specific coercive measure within a specific situation is morally acceptable, and if so, for which decisive reason. Both studies in ethics and empirical studies come with different and sometimes contradictive arguments. For studies in ethics, some describe moral arguments against the use of coercive measures such as the infringement on patient’s autonomy, human dignity and integrity [4–6]. Others justify the use of coercive measures because they might decrease the suffering and/or the risk for the patient, and improve patient autonomy on the long term [5, 6]. Reluctance to use coercion may even deny the patient the help and care that he/she sometimes desperately needs [7]. Others suggest that pro and contra arguments should be weighed: the use of coercive measures can be morally acceptable as long as “*the ‘benefits’ with regard to protection or treatment outweigh the ‘negative effects’ on patients’ autonomy, integrity and comfort*” [8] (p. 231). In a Dutch empirical-ethics study, eight normative guidelines with respect to when and how to use coercive measures in a right way have been developed [9, 10]. However, none of these studies focusing on moral arguments and normative guidelines give clear cut answers to whether the use of a coercive measure in a particular situation is morally justifiable or not.

In addition to this, there is no clear evidence on whether coercive measures are practically or clinically ‘helpful’ or ‘effective’ [11–15]. At the same time, many studies point to the harm caused by the use of coercive measures for both patients, their family members, as well as their care providers [16–24]. Other studies show that some patients in retrospect report that coercion was a ‘correct’ or even ‘necessary’ approach in a given situation [8, 25], and that patients and relatives even may blame professional caregivers for using coercive measures too little, too late or too short [26, 27]. Hence, despite the increasing empirical studies pointing towards the negative aspects of the use of coercion, it remains difficult to determine the moral justification of the use of coercion in particular situations.

### Normative attitude towards coercion

Partly due to this moral uncertainty, studies into the normative attitude of health care employees regarding the use of coercive measures have become more and more important [6, 28–30]. Some central questions remain unanswered: What is the right timing for the initiation and termination of the use of a coercive measure? Which measures should be tried first? What is an appropriate attitude when executing a coercive measure?

In order to find out how staff in mental health care normatively thinks about, conceptualize and justify the use of coercion, a questionnaire on normative attitudes towards the use of coercive measures in general was developed and validated in Norway: the Staff Attitude to Coercion Scale (SACS) [31, 32]. In this Norwegian study^1^, Husum and colleagues found three clinically meaningful and internally consistent dimensions (SACS subscales) related to staff’s normative reasoning: coercion seen as offending towards patients; coercion seen as needed for care and security; and coercion seen as a treatment intervention. The first subscale (‘offending’) contains critical statements towards using coercion in care. They emphasize the potentially offending, humiliating and negative effects of the use of coercion on both patients and the relationship between patients and staff. The other two subscales describe ‘positive’ aspects of the use of coercion in care. ‘Care and security’ has been described as a pragmatic view on coercion because the items in this subscale represent a view in which coercion is needed for the care and security of all involved [31]. The items in the third subscale ‘treatment’ give even a more positive impression of coercion, indicating that it is needed and useful for the treatment itself. These three different dimensions of staff’s normative attitudes on coercion in general seem to represent a certain kind of normative thinking about coercion that has also been described in other studies on staff attitudes towards coercion [28, 33].

### Moral doubt

Given the moral uncertainty whether, in the end, use of a specific coercive measure is morally right, we became interested in the concept of ‘moral doubt’. As working definition, we describe moral doubt as not knowing or being uncertain whether something is morally right or justified^2^. We wondered whether if and how often health care professionals in mental health care were morally uncertain in general (i.e. not related to a specific topic of theme) and regarding the use of coercion in particular, and whether these two kinds of moral doubt influenced their normative attitude towards coercion. Within the context of the use of coercive measures, a health care employee’s moral doubt regarding the use of coercion consist of asking oneself questions like: When is it right to use coercion? When are we too late or too early regarding the use of a coercive measure? Which coercive measure is better (or less worse) and/or what are appropriate alternatives? When is the use of coercion morally sufficiently justified and what kind of justification is needed (e.g. professional guidelines, evidence-based knowledge, normative guidelines)?

In general, one finds both critical and positive valuations of moral doubt. Within clinical practice moral doubt is not always valued positively. It can be seen as a sign of professional weakness, being unexperienced or not being decisive enough. This can lower moral perception and reflection, and discourage requests for (ethics) support when dealing with moral doubt [34]. Furthermore, asking questions about the moral rightness of moral justification of actions and decisions can be easily experienced as criticism towards colleagues or those responsible for decision-making. Moral doubt can also have a paralyzing effect: not making decisions or not taking any action (or taking actions too late). Within the context on mental health care, moral doubt can hinder timely decisions or actions which might increase the chance of critical or even dangerous situations for both patients and staff. Due to this critical valuation of moral doubt, it can be difficult to be open about one’s moral doubt.

Within the field of (clinical) ethics, moral doubt is often associated with the epistemic uncertainty within the domain of ethics itself: there seems to be no universal ‘evidence’ or knowledge source for determining the moral rightness of our actions. Within the field of (clinical) ethics, moral doubt is often considered as both ‘appropriate’ and ‘positive’. Appropriate since it fits with the inherent epistemic uncertainty within (clinical) ethics; positive since moral doubt usually involves an extra critical awareness of how people reason and justify certain behaviour, routines or policy’s. Applied to the context of the use of coercion within mental health care: experiencing moral doubt may prevent a too quick or automatic use of coercion. More generally, one may argue that any kind of practice improvement requires a certain level of doubt, e.g. the ability to acknowledge the possible existence of better alternatives, and that established practices are not necessarily the only right or best ways of doing things. In order to help health care professionals and patients with their moral doubt, clinical ethics support services (such as clinical ethics consultation or moral case deliberation) are being implemented in (mental) health care settings [35–38].

### This study

In this paper we present the results of a cross-sectional survey study among multidisciplinary employees of seven departments in three Norwegian institutions for mental health care. We wanted to find out mental health care professionals’ normative attitude towards coercion and how often they experience moral doubt in general and moral doubt related to the use of coercion in particular (i.e. *research question 1*). Furthermore, we wondered if and how (the lack of) experiencing moral doubt is related to staff’s normative attitudes towards the use of coercion (i.e. *research question 2*). Finally, we wanted to find out to what extend gender, age, professional background and other professional contexts (such as: ward type, leadership responsibility, experience with coercion, staff’s decisional responsibility towards the use of coercion, staff’s years of working experience) were associated with the staff’s normative attitude towards coercion and experience of moral doubt (i.e. *research question 3*).

## Methods

The questionnaire used for this study is part of a large mixed-methods study on ethics, ethics reflection groups and coercion (2011–2015). The study described in this paper took place as a baseline measurement prior to the implementation of moral case deliberation (or ethics reflection groups) as a kind of ethics support^3^.

### Study design

This paper describes a cross-sectional survey study in which we executed an explorative statistical analysis.

### Study sample

The study sample existed of the employees of seven wards from three mental health care institutions in the south of Norway (i.e. 2 acute wards, 1 rehabilitation ward, 1 ward for youth mental health care, 1 ward with geriatric mental health care, 1 ward for outpatient services, 1 ward for specialist psychiatric care). During this study, all of these wards used some kind of coercive measures. The employees consisted of various health care professionals (such as: nurses, auxiliary nurses, psychiatrist (including psychiatrists in training), psychologists), team leaders and management. Employees were invited to fill in the written questionnaire both face-to-face during team or ward meetings and by email by the local study coordinator (i.e. an employee at that ward) and the management. Temporarily staff and supporting staff did not participate in the study.

### Instrument

For the results described within this paper we used the following questionnaires:

#### Staff Attitude to Coercion Scale (SACS)

Staff’s normative attitude regarding the use of coercion was measured with SACS [31] (see Table 1﻿ below). The SACS concerns the use of coercion in general and includes formal, informal and experienced coercion. As described in the Introduction, the SACS consists of 15 normative statements on coercion and represents three subscales (see Table 1 for all statements): coercion seen as offending (6 items); coercion seen as needed for care and security (6 items); and coercion seen as a treatment (3 items). Each item was scored on a Likert scale ranging from 1 (strongly disagree) to 5 (strongly agree). During the analysis, we recoded each item so that higher scores represent more agreement. For each of the three subscales we calculated mean scores and used these as dependent variables in separate multivariate analyses. Mean scores on ‘Offending’ and ‘Care & security’ were calculated only if respondents had valid answers on at least 4 of the 6 items) and on ‘Treatment’ when each of the three items was answered validly. Reliabilities of the three subscales were satisfactory (Cronbach’s α’s: 0.67 for ‘Offending’; 0.71 for ‘Care and security’; 0.67 for ‘Treatment’).

**Table 1:** The 15 normative statements of the SCAS [31]

I Coercion as offending subscale
• Coercion could have been much reduced, when giving more time and personal contact
• Scare resources lead to more use of coercion
• Coercion violates the patients integrity
• Too much coercion is used in treatment
• Use of coercion can harm the therapeutic relationship
• Use of coercion is a declaration of failure on the part of the mental health services
II Coercion as care and security subscale
• For security reasons coercion must sometimes be used
• Coercion may represent care and protection
• Use of coercion is necessary as protection in dangerous situations
• For severely ill patients coercion may represent safety
• Coercion may prevent the development of a dangerous situation
• Use of coercion is necessary towards dangerous and aggressive patients
III Coercion as treatment subscale
• Patients without insight require use of coercion
• Regressive patients require use of coercion
• More coercion should be used in treatment

#### Moral doubt regarding the use of coercion

The frequency of moral doubt specifically about coercion was assessed by the question “*How often in the last twelve months did you experience a situation in which coercion could be at stake and in which you were uncertain about what should be done?*” Answer categories for this question were: never, once, up to eight times, every month, every week, and every day. In order to have groups of sufficient size, we combined ‘never and once’, and ‘every month, every week, and every day’ into single categories. We also asked “*How frequently did you experience a situation in the last twelve months in which you performed or decided upon a coercive measure?*” Answer categories were the same as for frequency of moral doubt about coercion. Never and once were combined.

Additionally, we computed a new variable ‘relative doubt about coercion’. This variable expressed how often respondents had had moral doubts about coercion in the past twelve months *relative to* how often they actually experienced the use of coercion. We divided respondents into three groups: those who more often experienced doubts about coercion than the number of times they were actually involved in coercive measures; those for whom the frequency of doubt was equal to the frequency of experiencing coercion; and those who had doubts about coercion less often than they actually experienced coercion. Dummy variables for these groups were used in the multivariate analyses.

#### Moral doubt in general

Three questions measured moral doubt in general (i.e. not specifically related to coercion): 1) “*During my daily work activities, I often reflect upon the question: Do I do the right thing?*”; 2) “*I reflect upon the way my values and norms influence my decisions and actions*”; and 3) “*One can never find just one answer to the question: What is the right thing to do*?” These items were rated on a Likert scale ranging from 1 (strongly disagree) to 5 (strongly agree) and were included as separate independent variables since factor analyses did not result in a reliable sub-scale.

#### Professional context

In addition to the institution and department where respondents worked (acute ward, rehabilitation ward, ward for youth care, geriatric care, ward for outpatient services, or ward for specialist psychiatric care), we also gathered information about their professional background and context. We categorized the respondents’ profession into: ‘psychologists’, ‘psychiatrists & related medical professions’ (e.g. psychiatrist in training, physician, chief-physician), ‘nurses & related professions’ (e.g. auxiliary nurses, milieu therapist, helping assistant), ‘management’ (unit team leader, ward manager, director), and ‘other’ (e.g. physiotherapist or occupational therapist). Further, we asked if they had decisional responsibilities regarding the use of coercion (yes/no) or leadership responsibilities towards employees (yes/no). We also asked how many years of work experience within mental health institutions they had had (categorized into 0–4 years, 4–8 years, 8–15 years and 15 years or longer). Finally, we included information on age and gender (1 = male, 0 = female).

#### Analytic strategy

First, we compared characteristics of the respondents who were excluded from the study sample based on incomplete scores (*n* = 43) with those that were included (*n* = 379). Group differences were calculated with independent *t*-tests or Chi-square tests. Second, we performed bivariate analyses, using each of the three SACS scales as dependent variables. We used *t*-tests (for dichotomous independent variables), ANOVA F-tests (for categorical independent variables), and linear regression models (for continuous independent variables or items measured on a Likert scale with five consecutive values). In some cases we further explored differences between pairs of subgroups with post-hoc tests using Bonferroni correction. Third, we used two multiple regression models to simultaneously assess the associations between moral doubt, experience with coercion and professional background and the three SACS scales. In the multiple regression analyses we included respondents with complete data for all independent variables only.

## Results

### Response rate

We received 422 questionnaires (response rate of 48%). Only respondents with valid scores on the three SACS-subscales (i.e. the dependent variables) were included in the study sample (*n* = 379). Because of this, 43 respondents were excluded.

### Descriptive analysis

With respect to the first research question we present the following findings from Table 2 (see below). When looking at respondents’ normative attitudes on coercion, on average, they were not so sure whether coercion should be seen as offensive or not (M = 3.13, SD = 0.54). With respect to the ‘care & security’ attitude, respondents on average did agree with the viewpoint that coercion is needed for care and security reasons (M = 4.12, SD 0.50). Respondents slightly disagreed that coercion could be seen as a treatment intervention (M = 2.58, SD = 0.65).

**Table 2:** Descriptive statistics on all variables (*n* = 379)

	Valid n	% or mean (SD)
Demographics		
Gender (% male)	374	40.1
Age (years)	373	
< 29	51	13.7
30–39	114	30.6
40–49	103	27.6
50–59	79	21.2
> 60	26	7.0
Attitudes towards coercion (SACS, 1–5)		
Offending	379	3.13 (0.54)
Care and Security	379	4.12 (0.50)
Treatment	379	2.58 (0.65)
Moral doubt (1–5)		
Reflect upon doing right thing	375	3.31 (0.99)
Reflect on own values & norms	378	4.07 (0.61)
There is no single answer re. what is right	377	4.27 (0.71)
Frequency of experiencing coercion (past 12 months)	369	
Never or once	92	24.9
Twice – 8 times	117	31.7
Every month	80	21.7
Every week	59	16.0
Every day	21	5.7
Frequency of doubt about coercion (past 12 months)	368	
Never or once	147	39.9
Twice – 8 times	169	45.9
> Once a month	52	14.1
Relative doubt about coercion	366	
Doubt less than experience	191	52.2
Doubt equal to experience	138	37.7
Doubt more than experience	37	10.1
Hospital and ward type (n/%)	353	
Hospital 1		
Geriatric care	15	4.2
Hospital 2		
Acute care	80	22.7
Community care	17	4.8
Youth care	51	14.4
Specialist care	64	18.1
Hospital 3		
Acute care	68	19.3
Rehabilitation care	58	16.4
Profession (n/%)	379	
Psychologist	27	7.1
Psychiatrist & related	29	7.7
Nurse & related	221	58.3
Others	95	25.1
Management	7	1.8
Decisional responsibility re. coercion (n/% yes)	120	33.0
Leadership responsibility (n/% yes)	56	15.0
Experience in mental health care	339	
0–4 years	92	27.1
4–7 years	83	24.5
8–14 years	93	27.4
15 years or over	71	20.9

Regarding the items representing moral doubt in general, respondents on average strongly agreed that there will never be a single answer to the question ‘What is the right thing to do?’ (M = 4.27, SD = 0.71). They also reported that they reflect upon the way their values and norms influence their decisions and actions (M = 4.07, SD = 0.61). They were relatively more ambivalent whether they reflect often about whether they are doing the right thing, during their daily activities (M = 3.31, SD = 0.99).

With respect to the experience of doubt about coercion in the past twelve months, 39.9% never or once had doubts, while 45.9% had doubted twice up to eight times, and 14.8% had doubts about coercion monthly or more often.

Regarding the relative doubt about coercion, more than half of the included sample had less often moral doubts about coercion than the frequency of actually experiencing coercion (52.2%), 37.7% had moral doubts about coercion as often as they experienced coercion, while 10.1% had more often moral doubts about coercion than they actually experienced coercion in the past twelve months.

### Bivariate analyses

Regarding the second research question, bivariate analyses showed the following findings concerning moral doubt in general (see Table 3 below). The more one was wondering whether one does the right thing, and the more one was convinced that there was no single answer to the question ‘What is the right thing to do?’, the more one thought that coercion is offending towards patients (b = .09, *p* < .01; and b = .08, *p* < .05 respectively; Table 3). Furthermore, the more one was thinking about how one’s own values and norms influence one’s decisions and actions, the less strongly one saw coercion as a form of treatment (b = −.12, *p* < .05).

**Table 3:** Bivariate associations between moral doubt, experience with coercion, ward and professional background with three attitudes towards coercion (*n* = 379)^a^

		Offending		Care & Security		Treatment	
	Valid n	B (s.e.)	mean*/β*	B (s.e.)	mean*/β*	B (s.e.)	mean*/β*
Moral doubt in general (1–5)							
Reflect upon doing right thing	374	.09 (.03)	.17**	−.04 (.03)	−.08	−.04 (.03)	−.06
Reflect on own values & norms	377	.03 (.05)	.04	−.06 (.04)	−.08	−.12 (.06)	−.11*
There is no single answer	376	.08 (.04)	.10~	.03 (.04)	.04	.02 (.05)	.02
Freq. of experiencing coercion (past 12 months)	369				**		
Never or once	92		3.17		4.03		2.63
Twice – 8 times	117		3.08		4.06		2.52
Every month	80		3.12		4.15		2.56
Every week	59		3.09		4.32		2.66
Every day	21		3.28		4.10		2.32
Decisional responsibility							
no	240		3.15		4.10		2.63
yes	120		3.10		4.15		2.47*
Freq. of moral doubt about coercion (past 12 months)	368						
Never or once	147		3.06		4.11		2.61
Twice – 8 times	169		3.15		4.11		2.55
> Once a month	52		3.24		4.15		2.47
Relative moral doubt about coercion	366		*		*		
Doubt less than experience	191		3.12		4.17		2.59
Doubt equal to experience	138		3.14		4.11		2.52
Doubt more than experience	37		3.38		3.93		2.54
Hospital and ward type	353		*		*		**
Hospital 1							
Geriatric care	15		3.00		4.18		2.56
Hospital 2							
Acute care	80		3.27		4.19		2.58
Community care	17		3.31		4.31		2.41
Youth care	51		3.10		3.98		2.33
Specialist care	64		3.26		4.04		2.46
Hospital 3							
Acute care	68		3.13		4.22		2.70
Rehab care	58		3.00		4.18		2.78
Profession^b^	372		**				*
Psychologist	27		3.49		4.00		2.25
Psychiatrist & related	29		3.17		4.13		2.46
Nurse & related	221		3.18		4.15		2.58
Others	95		3.08		4.10		2.67

^a^ ~ =*p* < .10, * = *p* < .05, ** = *p* < .01. For categorical independent variables these indicate an overall *p*-value for differences amongst all categories

^b^ The group ‘managers’ has been left out of this analyses due to small n (7)

Those who more often doubted about the use of coercion tended to perceive coercion slightly more as offending and less as treatment, but the group differences were not statistically significant.

Concerning relative doubt, those with a higher frequency of moral doubt about coercion relative to the actual frequency of experiencing coercion, saw coercion more strongly as offending (overall differences among groups F = 3.56, *p* < .05) and less as ‘care & security’ (F = 3.76, *p* < .05). Post-hoc analyses showed for both attitudes that the differences between ‘less than experience’ and ‘more than experience’ were statistically significant. No differences in treatment attitude among the relative doubt subgroups were found.

In addition, we explored whether moral doubt in general, moral doubt related to the use of coercion and ‘relative doubt’ differed by professions and ward types. We found statistically significant differences among professions in the proportion who doubted more than once a month about the use of coercion (F = 5.191, *p* < .01): psychologists 29.6%, psychiatrists and related professions 34.5%, nurses and related professions 12.6%, managers 0.0%, and ‘others’ 7.7% (i.e. physiotherapists, unknown professions, and those without professional education). Post-hoc tests with Bonferroni correction reveal significant differences between psychologists and ‘others’ (*p* < .05), psychiatrists and nurses and related professions (*p* < .01), and psychiatrists and ‘others’ (*p* < .01). This means that compared to ‘others’, a significantly higher percentage of psychologists were in doubt more than once a month. And compared to the nurses and ‘others’, a significantly higher percentage of psychiatrists were in doubt more than once a month. We found no significant differences between groups for the other moral doubt variables e.g. relative doubt.

With respect to the third research question, frequency of experiencing coercion was not associated with seeing coercion as offending and a form of treatment. However, we found statistically significant differences in seeing coercion as important for care and security among groups with different amounts of experience (F = 3.80, *p* < .01). Post-hoc analyses with Bonferroni correction showed two statistically significant differences: those who had experience with coercion every week agreed stronger that coercion can been seen as care and security than those who experienced coercion ‘never/once’ or ‘2–8 times’ the last 12 months (both p’s < .01).

Further, those with decisional responsibility for using coercion disagreed slightly more with seeing coercion as treatment than those without decisional responsibility (M = 2.47 versus M = 2.63 respectively; T = 2.08; *p* < .05). No significant differences were found for ‘offending’ and ‘care and security’ subscales.

Differences in attitudes towards coercion were found between wards (F = 2.39, *p* < .10 for offending; F = 2.24, *p* < .05 for care & security; F = 3.10, *p* < .01 for treatment). Although on average employees neither agreed nor disagreed with the statements that described coercion as offending, respondents from Community Care, Acute Care (Hospital 2) and Specialist Care seemed to agree a bit more that coercion could be seen as offending. On average, respondents from all wards agreed that coercion could be seen as care and security; Community Care most strongly. Finally, Community Care, Youth Care and Specialist Care disagreed more with statements that mentioned that coercion could be a seen as a treatment, even though the disagreement was still very moderate.

We found differences among professions in the offending subscale (F = 8.51, *p* < .01). These differences suggested that on average, psychologists slightly agreed more with seeing coercion as offending than the other professions (M = 3.49 compared to M = 3.08 to M = 3.17 respectively). We also found differences by profession for the treatment subscale (F = 3.41, *p* < .05): psychologists tended to disagree more with seeing coercion as treatment than the other professions (M = 2.25 versus M = 2.46 until M = 2.67), although the overall disagreement was still moderate.

Leadership responsibility, years of experience in mental health care, gender and age were not associated with attitudes towards coercion (therefore not shown in Table 3).

### Multivariate analyses

Since we found substantial differences in normative attitudes towards coercion amongst the various ward types, we adjusted the multivariate analysis for ward type (not shown in table). When looking at the three items of general moral doubt, the multivariate analysis showed that when adjusting for all other variables in the model, more reflection on what is the right thing to do was associated with a stronger agreement with seeing coercion as offending (b = .09, SE = .03, *p* < =.01) (see Table 4 below). The same applied to those who stated that there is no single answer to the question what is the right thing to do (b = .08, SE = 0.4, *p* = .06). For ‘treatment’, we found a marginally significant negative association between more reflection on values and norms with seeing coercion less as treatment (b = −.11, SE = .06, *p* = .07).

**Table 4:** Multiple regression model (only significant associations), regressing normative attitudes towards coercion on moral doubt, frequency of doubt and experience with coercion, leadership responsibility and profession (*N* = 328)^a^

	Offending		Care & Security		Treatment	
	B (SE)	Bèta	B (SE)	Bèta	B (SE)	Bèta
Doing the right thing	.09 (.03)	.16**				
Own values/norms					−.11 (.06)	−.11~
What is right	.08 (.04)	.10~				
Relative doubt	Reference group = frequency of doubt equal to frequency of experiencing coercion					
less than experience	−.00 (.07)	−.00				
more than experience	.24 (.10)	.14**				
Leadership					−.15 (.08)	−.11~
Profession^b^	Reference group = nurse & related					
Psychologist	.34 (.12)	.17**	−.20 (.11)	−.11~	−.31 (.14)	−.13*
Psychiatrist & related	.08 (.11)	.04	−.04 (.11)	−.02	−.02 (.14)	−.01
Others	−.07 (.07)	−.05	−.02 (.07)	−.01	.04 (.09)	.02
Adjusted R-square		.10**		.03*		.05**

^a^Only respondents with complete data were included. All models were adjusted for department/ward type and for reporting of two professions

^b^The group ‘managers’ has been left out of this analyses due to small n (7)

~ = *p* < .10, * = *p* < .05, ** = *p* < .01

Those with high relative doubt about coercion (more than experiencing coercion) stronger agreed with seeing coercion as offending (b = .24, SE = .10, *p* < =.01).

Marginally significant differences were found regarding differences between respondents with or without leadership responsibility: those with leadership responsibility disagreed stronger with seeing coercion as treatment (b = .−15, SE = .08, *p* = .07).

Differences between professional groups remained present in the multivariate analyses. Compared with psychiatrists and the group ‘others’, psychologists saw coercion more as offending (b = .34, SE = .12, *p* < =.01), less as care and security (b = .−20, SE = .11, *p* = .07), and less as treatment (b = .−31, SE = .14, *p* = .03).

The remaining associations found in the multivariate model were not statistically significant.

## Discussion

In this explorative cross-sectional survey study across seven wards in three Norwegian hospitals for mental health care, we studied the relationship between three kinds of doubt (‘moral doubt in general’, ‘moral doubt related to coercion’, and ‘relative doubt’) and the staff’s normative attitude towards the use of coercive measures, controlled for professional background and contextual features. We will first briefly summarize the results and then discuss more in detail some central findings.

With respect to the averages of staff’s normative attitude towards coercion (measured with the SACS questionnaire [31]), we found no (strong) critical attitudes regarding the use of coercive measures. The respondents (*N* = 379) neither agreed nor disagreed with normative statements which stated that coercion should be seen as offending, which could be understood as an collective expression of a kind of doubt. At the same time, they agreed that coercion is needed for care and security (especially those who are often involved in the use of coercion), and they only slightly disagreed with seeing coercion as treatment (especially those with decisional responsibility and leadership responsibility). Ward type showed significant differences regarding the normative attitudes towards coercion, especially respondents from Community Care, Acute Care, Youth Care and Specialist Care were more critical (as compared to Geriatric Care and Rehabilitation Care). Psychologists were most critical towards coercion.

Respondents did not often experience moral doubt regarding the use of coercion: 40% did not doubt at all or only once, and 46% doubted no more than 8 times in the last 12 months^4^. One explanation for this could be that employees who have more experience with coercion become more confident and less critical towards the use of coercion. When comparing the frequency of experiencing moral doubt regarding the use of coercion and the frequency of experiencing an actual situation in which coercion has been used (i.e. what we called ‘relative doubt’), 10% of the respondents experienced more often moral doubt than they were involved in situations in which coercion has been used. Two possible clarifications for this finding could be that 1) participants experienced doubt in situations where they finally did NOT use coercion, or 2) participants did not compare or remember the answers to both questions. Psychologists and psychiatrists relatively experienced most doubt regarding the use of coercion which might be caused by their decisional responsibility regarding the use of coercion, which especially apply to psychiatrists. With respect to the fact that psychologists experience most moral doubt, another explanation could be that psychologists are more trained to use a relational approach to patients while psychiatrists are more trained with using a medical framework in which diseases need to be treated and risks need to be prevented.

Experiencing moral doubt related to coercion was not statistically related with staff’s normative attitude towards coercion, but those who had a high relative moral doubt (related to how often they experienced coercion) perceived coercion more as offending and less as care and security.

With respect to experiencing general moral doubt, respondents did not agree nor disagree whether they often reflect upon whether they do the right thing. However, they reflect upon their values and norms, and they agreed that there will never be a single answer to the question ‘What is the right thing to do?’ The more staff experienced general moral doubt, the more one thought that coercion is offending and should not been seen as treatment.

### The SACS scores and related studies

Given the general understanding that the use of coercion is morally problematic, we were somehow surprised that, on average, the staff did not clearly agree that the use of coercion in general is potentially offending. This may be caused by not having a conscious opinion about the question whether coercion is offending or by not being aware of the possible offending elements of coercion. Perhaps the fact that coercion is often seen as care and security did prevent staff from acknowledging that it *at the same time* can be offending. Furthermore, it is possible that critical normative attitudes towards the use of coercion in mental health care to some extent can be experienced as potentially provocative or offensive towards staff. Finally, relatively low scores on the offending-subscale might also be related to the specific (wording of the) items of the SACS questionnaire and the fact that the SACS is about coercion in general and does not involve concrete descriptions of coercive measures.

The SACS questionnaire is used in several Norwegian populations of mental health care staff [31, 32, 39, 40] and most of the findings presented in this paper harmonize with findings in previous studies where staff also were uncertain whether coercion should be seen as offending and whether coercion harms the relation between staff and patients. An exception to this may be a Norwegian study by Wynn [6] where staff believed the use of restraint and seclusion violated patients’ integrity could harm the provider/patient alliance and could frighten other patients. Furthermore, in the Husum studies [31, 32, 39, 40], staff also considered use of coercion to be needed for care and security reasons. This again fits with the findings of Wynn [6] where violence, self-harm and threats were given as main reasons for the use of restraint. The fact that in our study particularly those with more experience of coercion agreed that coercion can be seen as care and security, also resembles Wynn’s finding that a majority of staff believed the restraint interventions were used correctly. Furthermore, this finding might refer to a stronger pragmatic attitude toward coercion when staff is more often involved in situations with coercion. In a Dutch study, Doeselaar and colleagues [28] indeed found that the more often staff had been involved in situations with coercion, the more pragmatic staff thought about the use of coercion. According to a Finnish study from Lind [41], it seems that “*Habituation to restraint and coercion brings with it acknowledgement of the necessity and desirability of the use of seclusion and it becomes a legitimate and justifiable practice*” (cited in Doeselaar et al. [28], p. 106).

With respect to the differences found among wards: this can probably be explained by different professional cultures of dealing with coercion but also by the existing variety among wards in which type of coercion is used and how often each type of coercion is used. The critical attitude of psychologists and the more pragmatic attitude of psychiatrists is also found in the study by Doeselaar et al. [28] in which they described three types of attitudes/groups: ‘transformers’ (on average: above 40 year, psychologists, more often involved in seclusion), ‘maintainers’ (on average: around 30 year, psychiatrists, monthly involved in seclusion), and ‘doubters’ (on average: under 30 year, most nurses, daily involved in seclusion).

### The complex relationship between normative attitudes, behaviour and moral reasoning

This study did not describe how staff’s normative attitude is related to the actual use of coercive measures. There is a long tradition of research which demonstrate an incongruence between what we think our belief system is, what our belief system actually is, and what we actually do [42, 43]. For example, based on the theories of ‘cognitive dissonance’, we may change our attitudes in retrospect to fit our behaviour [44]. A related Swedish clinical study in which psychiatrists’ normative attitudes towards the use of restraint and involuntary treatment were compared with their response to three vignettes, showed a gap between what they claimed to be their ethical beliefs and what “*… clinical experience dictates in practice*” [45] (p. 395). Hence, one should be careful in assuming that normative attitudes and moral doubts related to the use of coercion automatically inform us what respondents will actually do in clinical practice. Further research on the relationship between normative attitude, moral doubt and actual behaviour is needed in order to both a) understand the clinical implications of the studied normative attitudes and moral doubts, and b) develop ways to stimulate the critical reflection on the use of coercion in clinical practice.

In case one aims at developing a more critical attitude towards coercion among health care professionals, empirical and theoretical concerns how normative attitudes and moral judgments are constructed become important. Related research in moral psychology on moral judgment has long been dominated by rationalist models, in which moral knowledge and moral judgment are understood as being reached primarily by a process of reasoning and reflection [46, 47]. Hence, moral judgments related to coercion are thought to be logically caused by moral reasoning on coercion. However, based on empirical and theoretical research, Haidt [48] presented a social intuitionist model and gave 4 reasons for considering moral reasoning to be a post hoc construction generated after a moral judgment has been reached. The model is a social model in that it de-emphasizes private reasoning done by individuals and instead emphasizes the importance of social and cultural influences. Applied to our study, this might be an indication that moral judgement about the use of coercion is influenced by social processes and cultural influences in mental health care settings, instead of logical and rational reasoning processes. Stimulating critical moral deliberation in wards with multidisciplinary groups, in which the moral justification of the use of coercion is thoroughly scrutinized, may stimulate a more critical attitude towards the use of coercion without suggesting that the use of coercion is *never* morally justified.

### Moral epistemic uncertainty and moral doubt

In connection with moral judgements on the use of coercion it can be relevant to look more detailed at the concepts of epistemic uncertainty and its relationship with moral doubt. As mentioned briefly in the Introduction, we assume a moral epistemic uncertainty related to the fact that one can never find or know an universal moral answer to the question whether the use of coercion in particular situations can be morally justified. And even if one thinks that the use of coercion in particular situations is morally justified, the particular values and norms that are used to justify this use may differ. We also assume another kind of moral epistemic uncertainty, referring to the logical order of moral reasoning: the necessity and moral appropriateness of a specific act of coercion never automatically follows from the factual situation. Or, to put it differently, facts A do not automatically lead to the use of coercive measure B. Hence, opting for coercion always entails an implicit or explicit moral judgement, related to the staff’s specific interpretation and valuation of both the facts, the specific coercive measure, and the relation between facts A and coercive measure B.

These two kinds of moral epistemic uncertainties suggest that we would expect that most respondents should experience moral doubt regarding the use of coercion on a regular base, which was not confirmed by the findings of this study. However, as mentioned above, 40% of the employees experienced no or just one moment of uncertainty with respect to the use of coercion while half of the respondents doubted only between two and eight times. At the same time, most of the respondents agreed with the statement that there will never be a single answer to the question ‘What is the right thing to do?’ Apparently, stating that there is no single answer to the question ‘What is the right thing to do?’ does not automatically lead to often experiencing moral doubt when actually deciding about the use of coercion. Obviously, there is a gap between theoretically acknowledging whether there is always some kind of moral epistemic uncertainty and actually experiencing moral doubt. Furthermore, experiencing moral doubt might be related with a more general epistemic uncertainty – e.g. that the scientific evidence base for coercive practices is generally weak or that facts (e.g. risk/dangerousness) can be interpreted differently. Hence, it depends on many other factors whether moral doubt is actually experienced and expressed or shared.

Given the fact that the use of coercion is inherently morally problematic, it would be worthwhile to study more precise a) how decisions regarding the use of coercion are morally justified and with what kind of scientific evidence, b) whether this specific moral reasoning differs among professions, ward types and specific coercive measures, and c) whether the moral justification of the use of coercive measures changes over time.

One of the practical implications of this study is perhaps that employees, leaders and researchers could pay more positive attention towards the experiencing of moral doubt on coercion in mental health care. They could explicitly ask about moral doubt experiences and how experiencing moral doubt helps the team to think critically and creatively about the use of coercion.

### Ethics support: increasing or decreasing moral doubt?

Recent studies report that health care staff experience various ethical challenges related to the use of coercion [49–51] and that they lack specific time, methodology and expertise to deal with these ethical challenges [51]. Earlier implementation and evaluation studies found that clinical ethics support, such as moral case deliberation or ethics reflection groups, helped the health care staff to better deal with ethical challenges [36, 37, 52–57]. In moral case deliberation, a trained facilitator uses a specific methodology for moral reasoning in order to scrutinize specific decisions and actions, and to reflect upon ethical challenges from various angles. Follow-up research should find out whether the structural implementation of moral case deliberation further decreases or increases staff’s experienced moral doubt on coercion. Moral case deliberation is often only offered as a response to staff’s experience of moral doubt. Perhaps, moral case deliberation should also get implemented in order to stimulate and cause moral doubt. To some degree, one could argue that increasing moral doubt can be seen as something positive. For example, it can stimulate creative and critical thinking and it may increase staff’s curiosity for alternatives to coercion. Moral case deliberation might also increase the awareness of (implicit or hidden) moral challenges and improve the moral perception of “doubtful” or questionable practices. However, moral doubt may also complicate or delay decisions and actions (which may be good or bad), increase staff’s feeling of uncertainty or cause staff’s feelings of lack of competence. The question whether and for which reason moral doubts is a good thing and whether not, including the relation with ethics support activities, needs more attention in both clinical practice and research.

In the end, we think that every use of any coercive measure needs a critical normative assessment whether it is morally appropriate to use that specific coercive measure at that moment and in the way it is executed. Even if the use of coercive measures can be morally grounded in professional guidelines, legal regulations and ‘good’ intentions, and even when the use of coercion leads to ‘good’ consequences, using coercive measures always means limiting the freedom of persons by the use of institutional and professional power. Increasing moral doubt might require and stimulate (joint) reflection on what is morally right to do. Ultimately, critical reflection on the use of coercion is a prerequisite for the quality of care.

## Limits and strengths of the study

There are several limitations of this study. The data are cross-sectional; follow-up research within the same sample is needed. Given the European differences with respect to staff’s normative attitude towards coercion, this study is not automatically representative for Europe. Concepts and items of the SACS may be interpreted differently due to different national health care laws and cultural backgrounds. We therefore recommend additional studies in other European countries on moral doubt. This survey took place within (wards of) mental health care institutions of which the management and the teams had decided to implement moral case deliberation on the use of coercion (directly after this survey). We do not know whether this made the employees more critical towards or interested in thinking about coercion, and whether they were more aware about the possibility of being morally uncertain about coercion. Another limitation of this study is the fact that only normative attitudes towards coercion *in general* have been studied. In the end, details and context are needed in order to make a normative judgment about the use of coercion (as is being done in moral case deliberations).

The concept of moral doubt within this study is merely operationalised in an explorative way, further conceptual and qualitative research is needed. For example future research should elaborate more on several nuances with respect to the concept of moral doubt (e.g. difference between not knowing that p, being uncertain about p and doubting that p) and the differences between moral doubt and other kinds of doubt. Furthermore, it is not clear whether ‘reflecting upon’ resembles moral doubt and whether someone who first experienced moral doubt can become quite confident about his\her moral judgments in the end. With respect to empirical research, future research should find out on whether moral doubt automatically entails a critical attitude and what the actual consequences of experiencing moral doubt are (e.g. becoming passive, feeling paralyzed, following routines, becoming active).’. The clinical meaning of the significant differences, i.e. whether staff actually strongly disagrees with each other regarding their normative attitudes towards coercion, needs to be further explored.

## Conclusions

On average, the health care staff in this study saw coercion mainly as needed for care and security, was unsure whether coercion should be seen as offending, and slightly disagreed with coercion seen as treatment. They did not often experience moral doubt regarding the use of coercion, even though they agreed that there is no single answer to the question of what is morally right to do. Those who experienced more often moral doubt, both in general and related to the amount of experienced coercion (i.e. relative doubt), were more critical towards the use of coercive measures. The more staff is involved in situations with coercion, the more they thought that coercion could be understood as care and security. Especially psychologists were more critical about coercion. Those with decisional responsibility for coercion and leadership responsibility saw coercion less as treatment.

The findings in this explorative study point towards both conceptually and empirically interesting associations between moral doubt and normative attitude (towards coercion). Conceptual and empirical research is needed to further explore these associations. This might also address the normative question whether moral doubt should be stimulated or discouraged among health care professionals and what role ethics support (such as moral case deliberation) should play in this respect.

## Abbreviation

SACS: Staff Attitude to Coercion Scale

## Footnotes

1 In Norway formal coercion includes use of mechanical restraint, medication in order to sedate, isolation and physical holding. In addition, “shielding” is being seen as coercion which assembles close and observational nursing (i.e. being present all the time). The Norwegian mental health care act also allows for use of involuntary use of medication, involuntary admission and observation, and involuntary follow up outside the hospital (in England called community treatment order). See for a translated version of the Norwegian health care law on coercion: http://app.uio.no/ub/ujur/oversatte-lover/data/lov-19990702-062-eng.pdf

2 According to this working definition, moral doubt differs from doubt in general due to its specific focus on the moral rightness and/or moral justification of something (e.g. an action or a decision).

3 See for related publications from this larger study on coercion, ethics and ethics reflection groups in psychiatry: [36, 49–51, 58, 59].

4 There were few substantial differences between the respondents who were excluded because of too much missing data on the SACS scales (*n* = 43) and the included respondents (*n* = 379). In the included sample, there was a higher percentage of psychologists, psychiatrists and nurses, and a lower percentage ‘others’ (i.e. physiotherapists, unknown professions, and without professional education). No-one in the excluded sample reported to have leadership responsibilities, as opposed to 15.0% in the included sample (*p* < .01). Furthermore, included respondents reflected more on their own values and norms than the excluded respondents (M = 4.07, SD = 0.61 and M = 3.75, SD = 0.77 respectively; *p* = .05), and agreed more with the statement that one can never find a single answer to the question what is the right thing to do (M = 4.27, SD = 0.71 and M = 3.75, SD = 1.18 respectively; *p* = .01). ^5^ In an additional focus-group interview study, within the larger research project, we asked these health care professionals how they dealt with moral doubt and ethical challenges [50].
